# Efficient Object Detection Using Semantic Region of Interest Generation with Light-Weighted LiDAR Clustering in Embedded Processors

**DOI:** 10.3390/s23218981

**Published:** 2023-11-05

**Authors:** Dongkyu Jung, Taewon Chong, Daejin Park

**Affiliations:** 1 School of Electronic and Electrical Engineering, Kyungpook National University, Daegu 41566, Republic of Korea; wjdxyz@knu.ac.kr; 2 Carnavicom Co., Ltd., Incheon 21984, Republic of Korea; twchong@carnavi.com; 3 Department of Physics, Hanyang University, Seoul 04763, Republic of Korea

**Keywords:** convolution neural network (CNN), object detection, LiDAR sensor, point cloud, semantic detection

## Abstract

Many fields are currently investigating the use of convolutional neural networks to detect specific objects in three-dimensional data. While algorithms based on three-dimensional data are more stable and insensitive to lighting conditions than algorithms based on two-dimensional image data, they require more computation than two-dimensional data, making it difficult to drive CNN algorithms using three-dimensional data in lightweight embedded systems. In this paper, we propose a method to process three-dimensional data through a simple algorithm instead of complex operations such as convolution in CNN, and utilize its physical characteristics to generate ROIs to perform a CNN object detection algorithm based on two-dimensional image data. After preprocessing the LiDAR point cloud data, it is separated into individual objects through clustering, and semantic detection is performed through a classifier trained based on machine learning by extracting physical characteristics that can be utilized for semantic detection. The final object recognition is performed through a 2D-based object detection algorithm that bypasses the process of tracking bounding boxes by generating individual 2D image regions from the location and size of objects initially detected by semantic detection. This allows us to utilize the physical characteristics of 3D data to improve the accuracy of 2D image-based object detection algorithms, even in environments where it is difficult to collect data from camera sensors, resulting in a lighter system than 3D data-based object detection algorithms. The proposed model achieved an accuracy of 81.84% on the YOLO v5 algorithm on an embedded board, which is 1.92% higher than the typical model. The proposed model achieves 47.41% accuracy in an environment with 40% higher brightness and 54.12% accuracy in an environment with 40% lower brightness, which is 8.97% and 13.58% higher than the general model, respectively, and can achieve high accuracy even in non-optimal brightness environments. The proposed technique also has the advantage of reducing the execution time depending on the operating environment of the detection model.

## 1. Introduction

The technique of using convolutional neural networks to search for specific objects in a data frame is used in many fields. The type of sensor used depends on the data being explored. First, sensors that generate two-dimensional, flat data are typically cameras. Sensors that generate three-dimensional spatial data are used, such as radar and LiDAR. A LiDAR sensor fires multiple lasers into the space of the area you want to measure, and the time it takes for the lasers to reflect and return to the sensor gives you data on the projected area. The 3D data generated by this process is point cloud data, which is a representation of the points in space where the lasers are reflected [1]. The data collected by a 3D sensor has the physical characteristics of the measurement area. Because of these physical characteristics, 3D data sensors can be highly reliable in environments where it is difficult for camera sensors to recognize their surroundings, which are affected by ambient light conditions. However, 3D sensor data requires larger data sizes and complex convolutional operations due to the increase in one dimension compared to 2D sensor data [2]. In fact, the object detection algorithms based on 3D sensor data proposed in various studies so far have high accuracy, but require a high-end processing environment including graphic process units (GPUs) [3,4,5].

There are many applications that use convolution neural network (CNN)-based object detection where low process resource consumption is an advantage. In a processor environment with relatively slow processing speeds rather than high-performance processors, low data throughput has a significant impact on processing speed. Autonomous vehicles, which need to recognize objects and situations around them for autonomous driving, use object detection algorithms on sensor data [6]. Objects around the vehicle are identified through object detection algorithms to understand the external situation and control the vehicle accordingly. In this case, the Driving Control Unit, which is the center of the autonomous vehicle, needs to process various events that occur during driving in real time in addition to object detection. The overall resources a vehicle uses for control are limited, so reducing the resource consumption of autonomous driving algorithms across the board can have a significant positive impact on the vehicle system as a whole. Also, if you are performing continuous object detection from a fixed location [7], such as a surveillance camera, you do not need fast processing speeds, but instead need to perform the behavior for a long time. Since the absolute amount of resource consumption reduction is proportional to the performance time, reducing object detection resource consumption can have a significant positive impact on long-running object detection systems. However, it is a big loss to give up the physical information obtained from 3D sensor data by using 2D image-based object detection algorithms instead of 3D data-based object detection algorithms for a lightweight process [8]. Therefore, in this study, we propose a method to increase the accuracy of the existing 2D-based CNN object recognition algorithm through a physical data preprocessing layer of 3D data.

**Figure 1:**
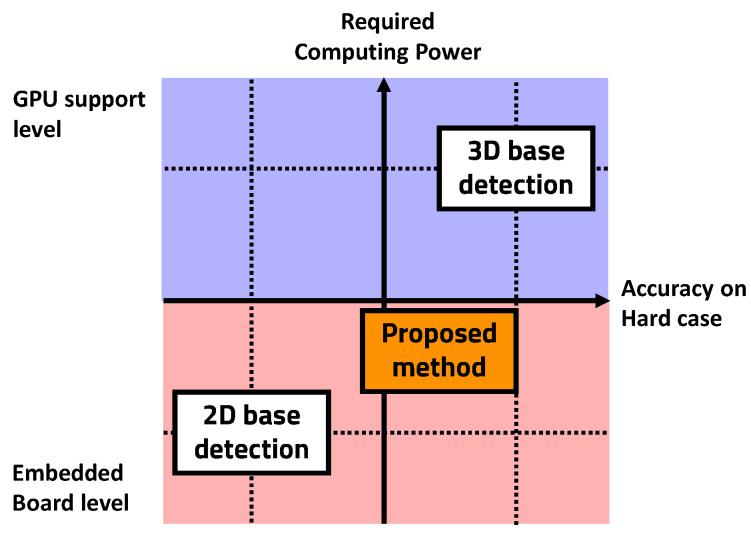
Computing power run on embedded board.

Different types of objects with physical volume have similar shapes, which can be described by different parameters. If the parameters can be selected that have unique values for different types of objects, they can be used to roughly classify objects with a simple algorithm. Using this same idea, region of interest (ROI) [9] can be generated in 3D data with physical properties where objects are likely to be present. The generated ROI can be used to build a lightweight object detection algorithm system that includes the advantages of 3D data using 2D-based object detection algorithms. By processing 3D data with only relatively low-computation algorithms, this method not only reduces the system’s resource consumption, but also lightens the hardware on which the system runs. In this paper, we propose an object recognition auxiliary layer that processes 3D data on an embedded board without using an external GPU and a 2D object recognition system using it. The proposed method can be driven with only additional computation on an embedded board that can drive a conventional 2D-based CNN object detection algorithm (Figure 1).

Many studies using conventional 3D LiDAR data and 2D image data present a special structure and process the two data in different ways according to the structure. In this process, 3D data and 2D data can be used simultaneously in the same processing step, or they can be processed in independent steps, but they are still affected by the abstracted other data. Although this method can show higher performance than existing object recognition methods that use each data separately, they are independent methods that are difficult to combine with other methods due to their special structure. Instead, in the method proposed in this study, the added 3D data processing layer operates completely independently of the 2D object recognition process, so that their respective algorithmic structures do not affect each other. As a result, the object recognition algorithm that performs post-processing can be replaced by another algorithm that performs the same function, resulting in flexible characteristics that satisfy various behavioral situations. Systems that use embedded processors require different amounts of resources depending on the environment in which they operate, and the embedded processor is selected based on the resources required by the system. Therefore, the flexibility that can be applied to different algorithms is a characteristic that is highly advantageous for use in embedded environments.

## 2. Related Research Work

### 2.1. ENet Masking + 2D Image Detection on Embedded Board

**Figure 2:**
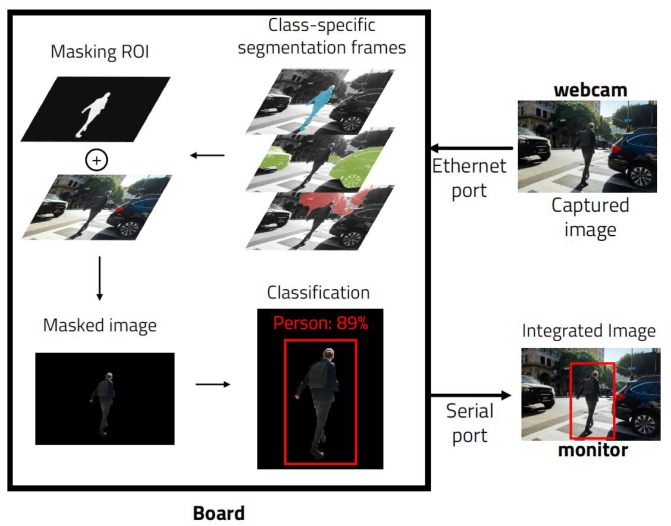
ENet masking + 2D image detection on embedded board.

A number of studies have been conducted to improve 2D image based algorithms through additional processes [10,11]. We performed a study to assist the 2D based CNN detection algorithm and improve its performance through an ENet algorithm that performs class-specific segmentation on 2D images [12]. This method, which uses two deep learning models, consists of generating filters for object detection through segmentation and performance of object detection on masked images (Figure 2).

At this time, separate deep learning algorithms are used for the two processes. First, a segmentation to identify the class is performed using ENet, and then an ROI masking filter is generated based on the result. After that, object detection is performed through several 2D image detection algorithms, including YOLO, on the image masked by the corresponding filter. The result is that instead of using more memory, we have a shorter processing time with higher accuracy and less computational quantities compared to general algorithms. Through this, a more effective object detection model is obtained on a lighter embedded board.

### 2.2. LiDAR Data Transmission Reduction for Light-Weighted Signal Processing Platform

**Figure 3:**
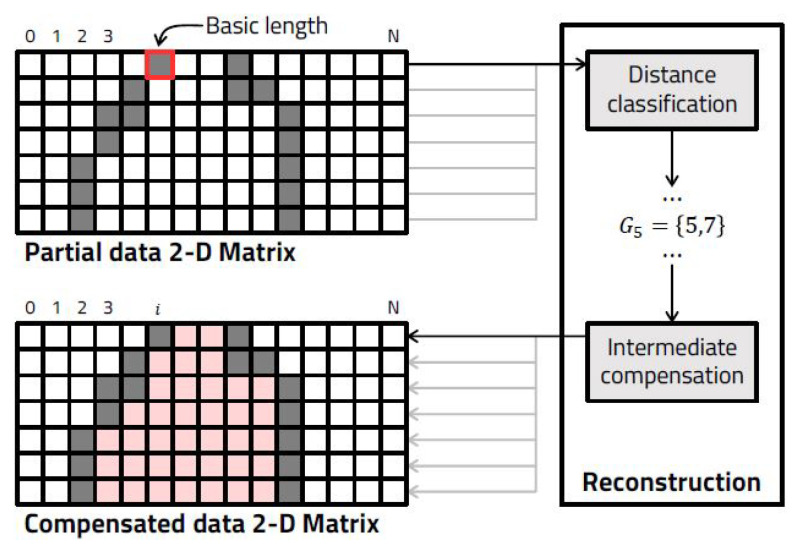
Concept of the point cloud change in the distance grouping step for each channel.

LiDAR sensor data generate a large amount of data in a single scan. This creates difficulty in systems that receive and process data in real time in lightweight embedded systems. We perform a study assessing a platform based on semantic depth data based data reduction and reconstruction algorithms that reduce the amount of data transmission and minimize the errors between original and restored data on a lightweight embedded board [13]. In the part that transmits LiDAR sensor data, the newly generated frame is compared with the immediately preceding frame in order to update an area in which a change amount exists to generate a transmission frame. In the system that receives these transmission frames, similar data are grouped and then reconstructed through convolution operations (Figure 3).

Through this, the total data transmission amount was reduced by an average of 87.4%, and the data transmission time was reduced by an average of 80.44%. Using this, LiDAR sensor data could be used with less load in a lightweight embedded system.

## 3. Semantic Object Detection Using Physical Properties

The object detection model proposed in this paper has a structure that acquires the physical characteristics of objects within the measurement range from three-dimensional point cloud data and performs a layer of semantic detection of those objects before the CNN object recognition algorithm [14]. The semantic detection layer converts the three-dimensional location information of the object that the system wants to track into two-dimensional location information, generates ROIs based on physical characteristics, and passes them to the object recognition algorithm so that the two-dimensional-based object detection algorithm can perform the final object detection within the area.

**Figure 4:**
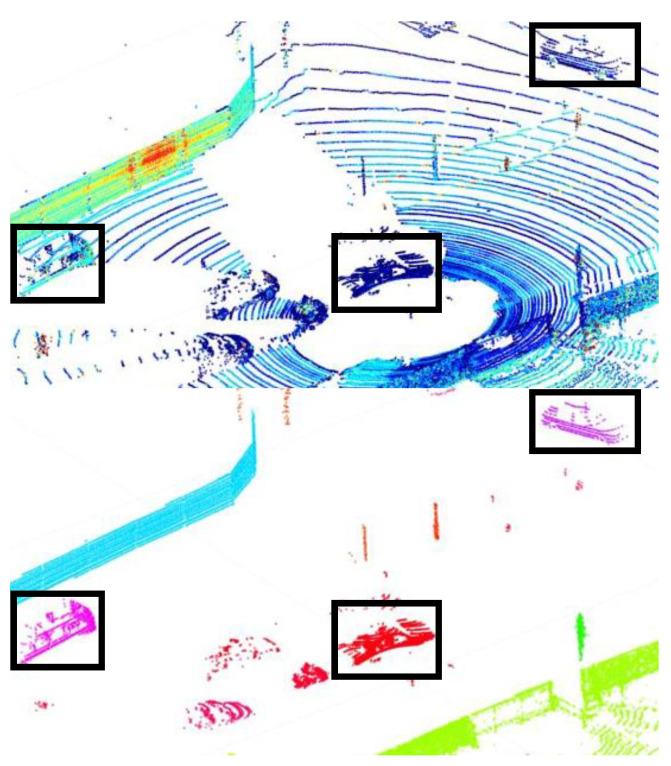
Object-clustered points.

Point cloud data are collected by LiDAR sensors, which store the three-dimensional coordinates of points where a laser fired from a LiDAR sensor is reflected. In other words, each point in the point cloud data means that an object that reflects the laser is physically present at that location. In this case, if an object with a certain volume is within the LiDAR sensor’s field of view, there will be adjacent points in the area occupied by that object. Areas with no points between adjacent groups of dots are empty spaces within the sensor’s measurement range where the laser did not reflect and return. Therefore, by separating groups of adjacent points into clusters, it is possible treat the set of points separated into one cluster as an independent object with a physical volume (Figure 4).

Each point cloud object separated by clustering has physical information about that object. This information includes things like distance from sensors, configuration, number of points, and density. These data, or the relationships between them, can also be unique features of the type of target object that we want to detect. The physical features considered in the semantic detection layer proposed in this paper are [distance from the sensor–number of points] and [bottom area of the bounding box–height of the bounding box].

**Figure 5:**
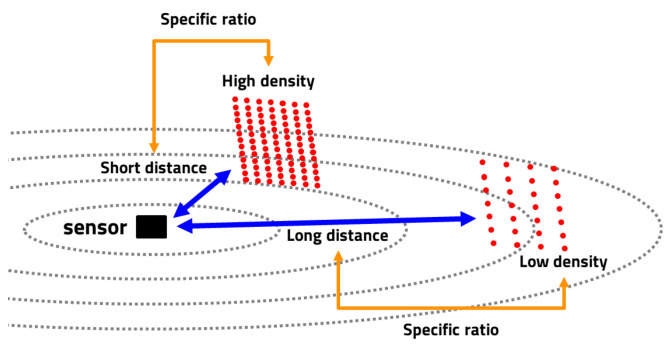
Relation with the distance from the sensor and the number of points.

The lasers fired by a LiDAR sensor are organized into horizontal and vertical channels, which radiate from the center of the sensor. When the same object is within the measurement range, the angle that it occupies in the sensor’s radiation range decreases as it moves away from the sensor. As a result, the number of lasers reflected by the object decreases, resulting in fewer points in the generated point cloud data. In other words, the distance between the object and the sensor is inversely proportional to the number of points in the point cloud. The relationship between distance and number of points is affected by the size of the target object. Even if objects of the same type are at different distances from the sensor, the ratio of the number of points to the distance gives you a similar value regardless of distance. Even if objects of different sizes are located at different distances and have the same number of points, their relationship to distance allows you to distinguish between them. Thus, the relationship between distance and number of points is affected by the size of the object and can be a unique characteristic of certain object categories (Figure 5).

**Figure 6:**
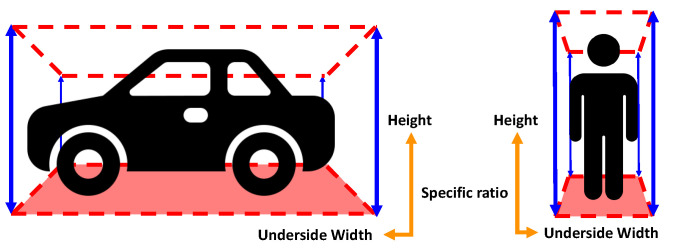
Relation with width of x-y plane and height.

Because point clouds exist in a three-dimensional region, each isolated point cloud object has a three-dimensional shaped bounding box region represented by the minimum and maximum points on each axis. The shape of the bounding box varies from object to object, but can have similar shapes depending on the type of object [15]. For example, the bounding box for a vehicle object is mostly a flat box, while the bounding box for a pedestrian object is more like a tall column. Objects with morphologically similar shapes like this have relatively similar bounding box shapes, which can be used to semantically categorize point cloud objects. For the x- and y-axis values, it is difficult to use them as unique values because of the different rotation angles of the objects with respect to the ground, which is the x-y plane (Figure 6).

Therefore, the semantic detection layer performs pseudo-detection using the proportionality constant between the width of the base of the bounding box, which has the same value regardless of the rotation state of the x-y axis, and the height, which is the z-axis value, as a unique physical property of the object.

## 4. Proposed Model

**Figure 7:**
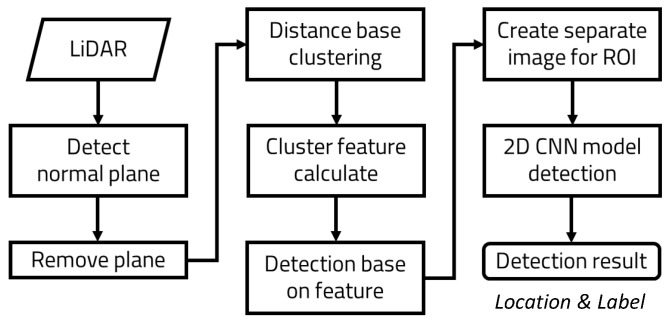
Full execution flow of the proposed process—sequence of actions for removing ground data, clustering, semantic classification of 3D data, and final object detection.

The process sequence of the object detection model proposed in this paper is as follows (Figure 7).

It performs preprocessing to perform clustering of point cloud data collected and transmitted by LiDAR sensors. First, it removes ground data that interferes with clustering. This process consists of searching for and removing removable ground within the data. It finds points inside the point cloud that constitute a removable-sized ground surface. Then, it finds and removes the data that it detected. This process is performed iteratively until there are no more removable-sized grounds that have been removed. After the ground data are removed, the point cloud data are clustered based on the distance data of each point. This separates the point cloud data of objects into groups of individual objects. From the points in these object groups, we extract the preset physical attribute values for classification. The extracted physical attributes are fed into a pre-trained classification model using machine learning. From the input attributes, we obtain a probability value that predicts what kind of object the object is. This probability value is used to semantically label the objects in the data in the first place. Obtain the location coordinates and area of labeled objects in the 3D region that have a high probability of being the target of the search. Convert the object locations in the 3D region to 2D image regions. Generate new 2D frames with the converted 2D regions and synthesize them with the 2D images to create ROI images. The generated image is then passed to the 2D-based CNN object detection algorithm. After receiving the 2D frame, the object detection algorithm performs object detection on the image in the area inside the frame to finally determine which objects are inside the frame. As a result, the location of the detected object inside the image and the label of the object can be obtained. See Algorithm 1.
**Algorithm 1:** Proposed Method    **Goal:** Create Region of Interest      1:PC = load_pointcloud(“LiDAR path”)      2:%Remove ground data      3:MaxDg = max_distance_of_ground_points      4:MaxADg = max_angular_distance_of_ground_points      5:MinSg = min_size_of_ground_points      6:Plane = RANSAC(PC,MaxDg,MaxADg)      7:**while** size(Plane) < MinSg **do**      8:    PC = select_points(PC,Plane)      9:    Plane = RANSAC(PC,MaxDg,MaxADg)    10:**end while**    11:%Clustering    12:MaxD = max_distance_of_points    13:MinSc = min_size_of_cluster    14:Cluster_list = cluster_list    15:UnCp = kdtree(PC)    16:**while** size(UnCp) > 0 **do**    17:    cluster = select_point(UnCp)    18:    (point,dist) = nearest_distance_point(cluster)    19:    **while** dist < MaxD **do**    20:        merge(cluster,point)    21:        remove(UnCp, point)    22:        (point,dist) = nearest_distance_point(cluster)    23:    **end while**    24:**end while**    25:%Extract feature + Semantic detection    26:Feat = struct(Cluster_list.size)    27:Model_1 = load_model(“Model path 1”)    28:Model_2 = load_model(“Model path 2”)    29:calib = load_data(“calibration path”)    30:**for** *i* ∈ Cluster_list.label **do**    31:    Feat(i) = extract_feature(Clusterlist(i))    32:    class_1 = class(Model_1, Feat(i).distance, Feat(i).density)    33:    class_2 = class(Model_2, Feat(i).width, Feat(i).height)    34:    **if** class_1 && class_2 **then**    35:        locate2D = convert3Dto2D(Cluster_list(i),calib)    36:    **end if**    37:**end for**

### 4.1. Remove Ground Data

**Figure 8:**
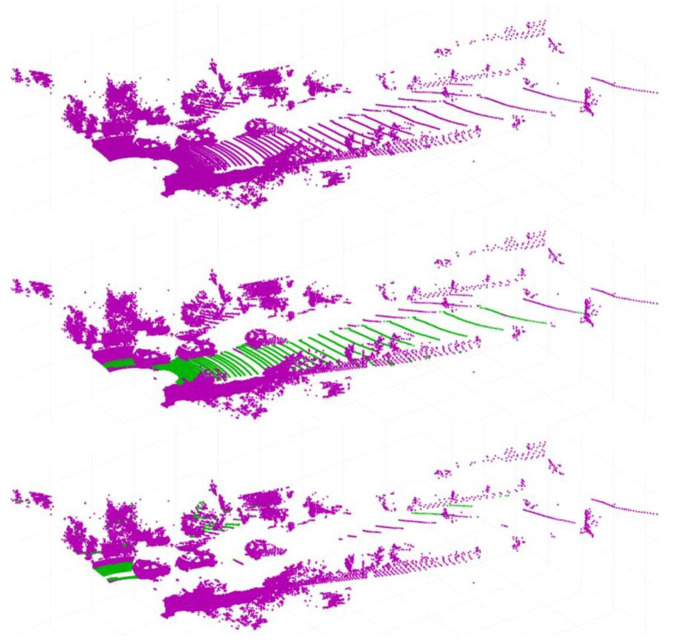
Ground removing process—origin data/find first largest plane and remove/find second largest plane and remove.

LiDAR sensors with a wide range of vertically oriented channels also collect data from the ground. Clustering for object detection distinguishes individual objects by the distance between neighboring points, but if ground data are present in the point cloud, all objects leading to the ground are recognized as one object. Therefore, before performing clustering, preprocessing is performed to remove ground data from the point cloud data. The semantic detection layer uses a method that looks for and removes the largest planar form of point data within the data. Since the ground is usually the largest planar object in the measurement area, we can treat a plane above a certain size as the ground. Therefore, the ground data are removed by iteratively performing the process of finding and removing the largest plane until the size of the largest plane present in the point cloud data are less than a certain size (Figure 8).

In the process of searching the ground, the range of the normal vector of the plane is set, and a plane horizontal to the ground may be excluded from the search object. In this paper, we explore the plane of the point cloud using the basic RANSAC algorithm [16].

### 4.2. Clustering

**Figure 9:**
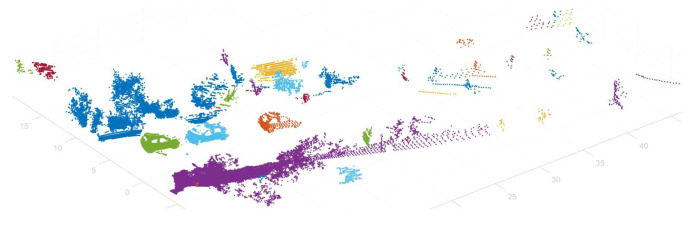
Result of the clustering process—points with the same color are one individual object.

Each point in point cloud data has a 3D coordinate value. In LiDAR data, the origin of the point cloud data are the location of the sensor, so the distance between each point and the sensor can be calculated as the sum of the squares of each coordinate value. Since the point data that make up an object are points of the same object, their distance values from the sensor are similar [17]. Therefore, clustering is performed to group points with similar values using the coordinate data of each point and the distance data from the sensor (Figure 9).

The clustering process performs clustering using the most basic clustering algorithm, the Nearest Neighbor Algorithm. In this case, clustering is performed by arranging the data into a 3D kd-tree [18] structure. The result is that the points corresponding to each object in the point cloud are grouped and separated. This type of clustering works well when each object is separated by some distance, but when the center points of two objects are very close together, it is hard to expect smooth behavior.

### 4.3. Semantic Classification

The physical attributes used for classification are obtained from each object separated in the clustering step. The physical properties used in the layer proposed in this paper are the distance between the center point of the object and the sensor, the number of points, the width of the floor, and the height data. To obtain these values, we calculate the hexahedral area of the object by finding the maximum and minimum *x*, *y*, and *z* values of the points contained in the isolated object. Set the distance between the object and the sensor based on the point closest to the center of the obtained hexahedron area, and set the area and height of the hexahedron floor as the values of the object. We semantically classified the object using a classification model that was trained on the object’s properties in advance. The classification model we used is a model trained by a machine learning algorithm based on a fine-grained K-Nearest Neighborhood (KNN) [19] algorithm.

(1) $$\left[\begin{array}{c} y_{1}\\ y_{2} \end{array}\right]=\left[\begin{array}{c} \mu_{1}p_{1}+\sigma_{1}p_{2}\\ \mu_{2}p_{3}+\sigma_{2}p_{4} \end{array}\right]$$

(2) $$y_{1},y_{2}\in Y_{truth}$$

We obtain a simplified linear relational coefficient $a_{n}$, $b_{n}$ using four parameters $p_{n}$ used for semantic detection (1). Semantic classification of the object is performed by determining whether the results of the two relational expressions are in the result range $Y_{trust}$, including an acceptable range (2).

**Figure 10:**
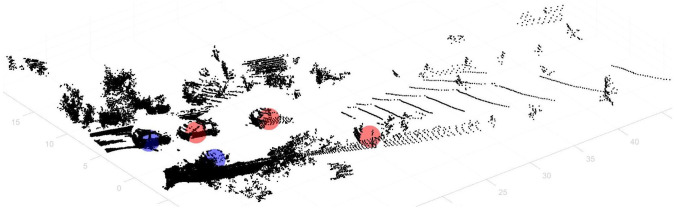
Result of a semantic classification process on point cloud data—the red regions are predicted target objects with a high probability, and the blue regions have a relatively low probability.

Through this process, the target object is not detected accurately, but preliminary targets that are thought to be the target object are found (Figure 10). The objects that are roughly determined to be objects by the classification model are finally determined to be objects by a CNN object detection algorithm based on 2D images. To do this, the positions and hexagonal regions of the initially classified objects in the 3D region are converted to positions and square regions in the 2D region.

(3) $$\left(u,v,1\right)=\left(\begin{array}{cccc} f_{u} & 0 & c_{u} & -f_{u}b_{x}\\ 0 & f_{v} & c_{v} & 0\\ 0 & 0 & 1 & 0 \end{array}\right)\cdot \left(x,y,z,1\right)$$

**Figure 11:**
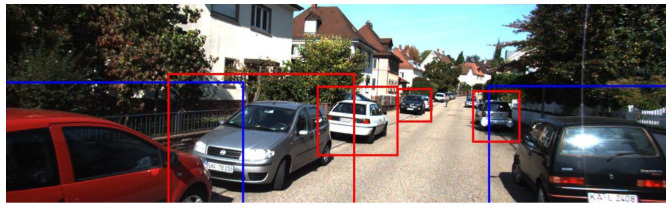
Result of the classification process on the image—the red regions are predicted target objects with a high probability, and the blue regions have a relatively low probability.

The 3D coordinates (*x*, *y*, *z*) are converted to 2D coordinates (*u*, *v*) via projection vectors based on the camera and LiDAR sensor information in the dataset (3). The converted 2D object regions are overlaid with the image regions from the camera collected simultaneously with the LiDAR sensor to indicate where the 2D object detection algorithm should perform (Figure 11).

### 4.4. Detection on 2D CNN Algorithm

**Figure 12:**
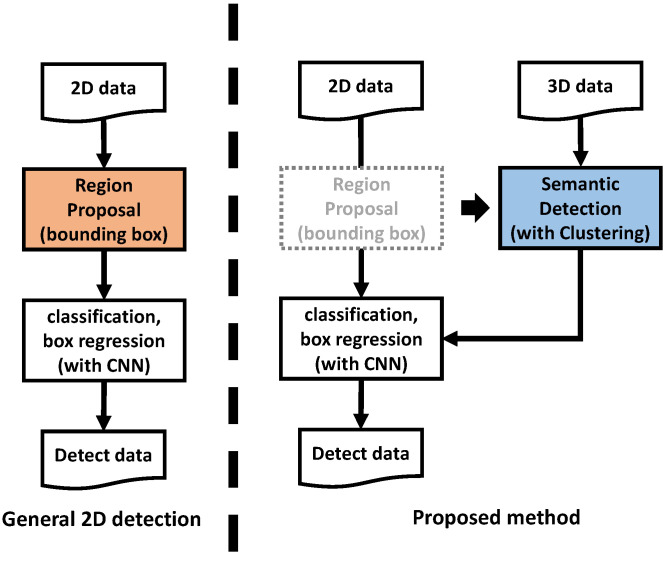
Detecting process compared with a 2D CNN algorithm—Replace region proposal with semantic detection.

There are two key processes in CNN-based 2D detection algorithms: the region projection process and classification. A 2D image-based CNN object detection algorithm handles these two processes in different ways and in a different order. In the previous step, the ROI generated by the projection tells us that there is an object in the region that we want to detect with a high probability. Therefore, the 2D CNN algorithm that performs the final object recognition in the last step can skip the process of detecting the objects to be recognized in the entire region. In other words, it can skip the region projection process of a traditional 2D CNN algorithm and perform object recognition inside the ROI. Therefore, the object tracking algorithm used in this thesis performs the object classification process within the ROI passed by the previous layer. The ROIs were used to separate the image into images of individual objects. The 2D detection algorithm performing the object detection was performed with the setting that there is one target object in the input image having the size of the ROI (Figure 12).

## 5. Experiments

**Figure 13:**
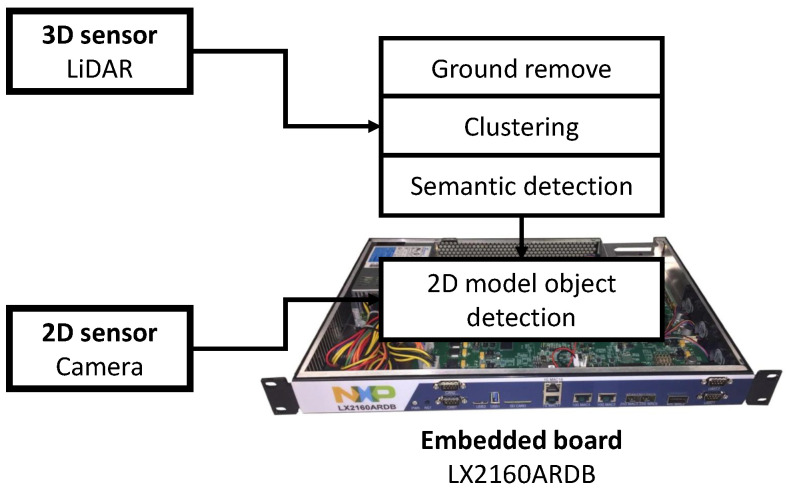
Environment for the proposed model.

The environment used in the experiment is as follows. We used the 3D object dataset of the KITTI dataset [20,21] for comparison of the model’s performance. The KITTI dataset consists of LiDAR point cloud data collected on a road with a Velodyne LiDAR Scanner and image data taken of the same area. The data were collected from outdoor roads, which makes it easier to remove ground data and allows for better clustering. The final stage 2D based object detection algorithm of the proposed model used YOLO v5 [22]. We performed the proposed algorithms and existing 2D based algorithms through clustering of LiDAR point cloud data and semantic object detection on the same embedded processor (Figure 13).

We used NXP’s LX2160ARDB development board as an embedded processor. The processor consists of 16 Cortex-A72, 2.2 GHz cores. We performed ground data removal and clustering using the PCL library [23]. In this experiment, we limited the target object to be tracked to “vehicle”, among various objects. See Table 1.

We used YOLO v5 as the 2D detection algorithm. The semantic detection layer of the proposed algorithm first separates the detected objects into individual images through semantic classification and passes them to the YOLO algorithm. At this time, we designed the YOLO algorithm for post-processing to omit the box prediction process by fixing the number of objects and box size inside the image. In other words, YOLO performing 2D detection omits the process of tracking the bounding box while knowing that there is one object inside the input image and its size is the entire image. In addition, since the image size of the separated individual objects was reduced compared to the original, we were able to reduce the image size of the YOLO algorithm by 1/16 to achieve performance improvement.

**Figure 14:**
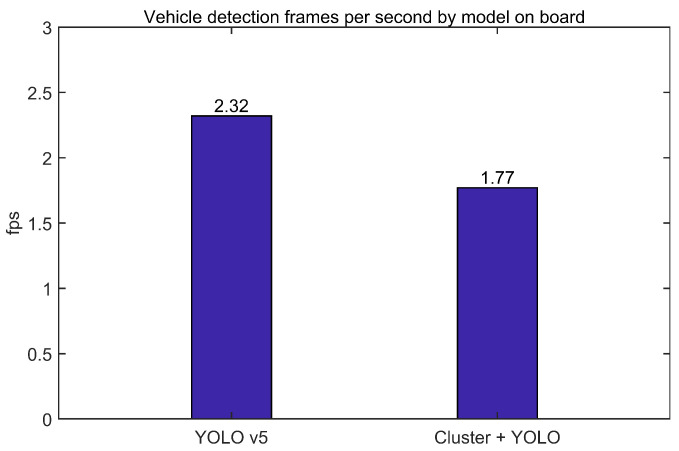
Detection frames per second by model on an embedded board.

The graph of Figure 14 is the frames per second (fps) of two models. First, we processed 2.32 fps for YOLO v5, a 2D based image detection algorithm, and 1.77 fps for YOLO v5, which performed the proposed clustering. The proposed model added time to perform clustering and semantic classification in advance, and because it performed individual object detection for each divided image frame, the performance time increased by about 23.7% compared to only YOLO.

**Figure 15:**
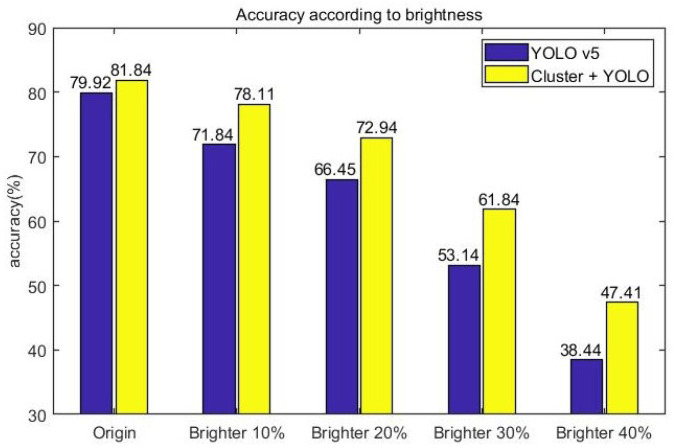
Accuracy according to brightness.

Figure 15 is the result of vehicle detection accuracy for image data modified to assume over-exposure situations, which increased the brightness of the original data and image data of the two models’ KITTI sets. In the original data, the only-YOLO-v5 model had an accuracy of 79.92%, and the proposed model had an accuracy of 81.84%, which was 1.92% higher than the only-YOLO-v5 model. As the brightness of the image data increased, the accuracy of the two models decreased. The only-YOLO-v5 model showed a 41.48% decrease in accuracy to 38.44% when the brightness was increased by 40% from the original image accuracy of 79.92%. The proposed model showed a 34.53% decrease in accuracy to 47.41% when the brightness was increased by 40% from the original image accuracy of 81.84%. When the brightness of the image was increased by 40%, the proposed model showed 8.97% higher accuracy than the only-YOLO-v5 model. In brightness environments above 40%, neither model showed significant recognition results, so we only set the target brightness up to 40% in this experiment.

**Figure 16:**
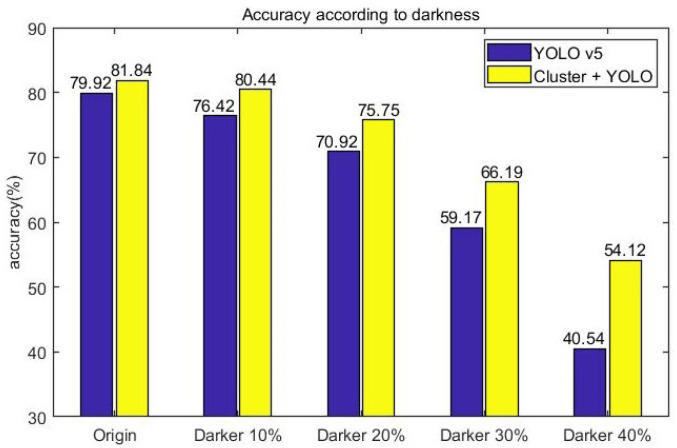
Accuracy according to darkness.

Figure 16 is the result of vehicle detection accuracy for the image data modified to assume a low-light situation, which reduced the brightness of the original data and image data of the two models’ KITTI sets. In the original data, the only-YOLO-v5 model had an accuracy of 79.92%, and the proposed model had an accuracy of 81.84%, which was 1.92% higher than the only-YOLO-v5 model. As the brightness of the image data decreased, the accuracy of the two models decreased. The only-YOLO-v5 model showed a 39.38% decrease in accuracy to 40.54% when the brightness was reduced by 40% from the original image accuracy of 79.92%. The proposed model showed an accuracy reduction of 27.72% to 54.12% when the brightness was reduced by 40% from the original image accuracy of 81.84%. When the brightness of the image was reduced by 40%, the proposed model showed 13.58% higher accuracy than the only-YOLO-v5 model.

**Figure 17:**
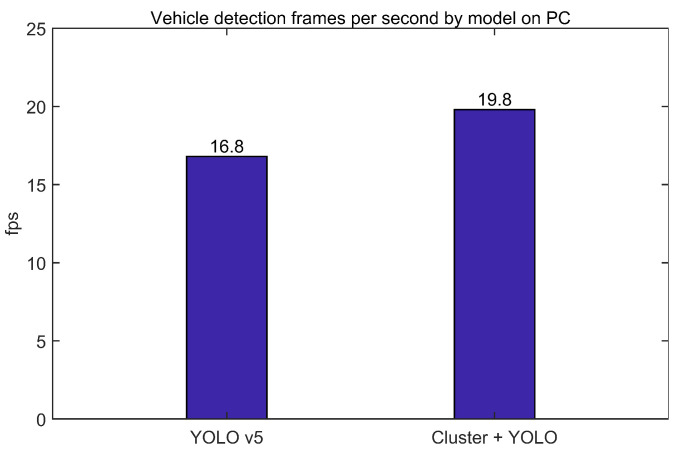
Detection frames per second by model on PC.

To check the performance difference according to the processor, we measured the processing time of the two models in the PC environment (Figure 17). We performed the experiment without a GPU in an Ubuntu 18.04 PC environment with 8 GB ram on an AMD Ryzen 54500U processor. Because we used the same detection model, there was no significant difference in accuracy performance. First, for YOLO v5, a 2D based image detection algorithm that performed the proposed clustering, we processed 16.8 fps and 19.8 fps for the proposed model, including clustering. Unlike the embedded model, the processing speed of the proposed model increased by about 17.8%. This is because the reduced processing time on the PC due to the omission of region exploration and the reduced image size to be explored is greater than the added processing time during the clustering process. Therefore, the proposed model shows different time performance trends depending on how long a particular process handles the clustering process and how long a particular region of the 2D detection model is performed.

**Figure 18:**
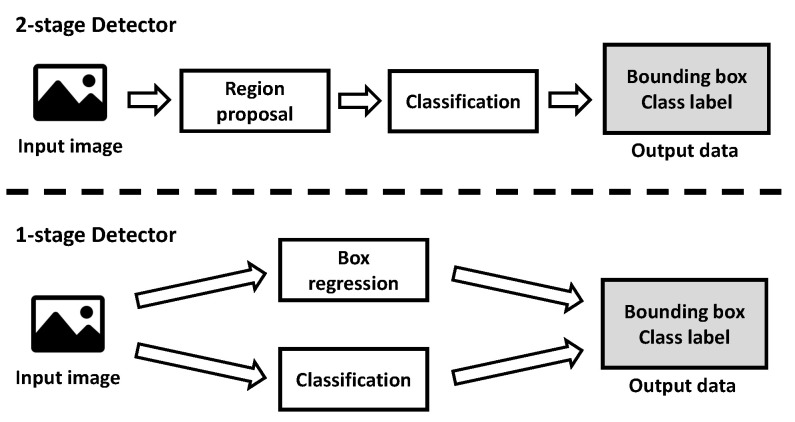
Two types of structures in the detection model.

The 2D-image-based CNN detector was largely divided into two types of structures as shown in Figure 18. The two-stage detector goes through a region proposal process to find an area that is presumed to be the object to be searched in the input image, and then it performs a classification process to distinguish the type of object in the area and to obtain the final object detection result. The one-stage detector processes two simultaneously performed processes on the two-stage detector in parallel to obtain the final detection result. Yolo v5, the model used in the paper, is a one-stage detector.

**Figure 19:**
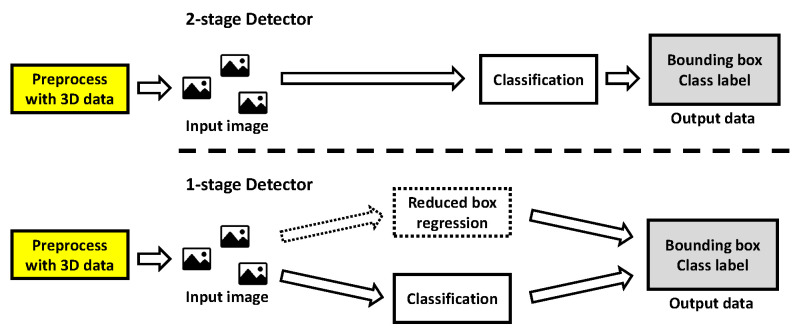
Two types of structures in the proposed detection model.

The proposed model replaced the region proposal/box regression process during the corresponding process. Figure 19 shows two types of detector structures to which the proposed model was applied. The box regression of the one-stage detector was replaced by a reduced box regression that acquires the size and location data of the input image and outputs them as they are. On the other hand, the region proposal of the two-stage detector was completely omitted, and classification was performed directly on the input image. The model used in the experiment was the same one-stage detector SSD [24] and the two-stage detector Fast R-CNN [25] for comparison with Yolo v5, which is the one-stage detector. The two models have the exact characteristics of a one-stage detector and a two-stage detector.

**Figure 20:**
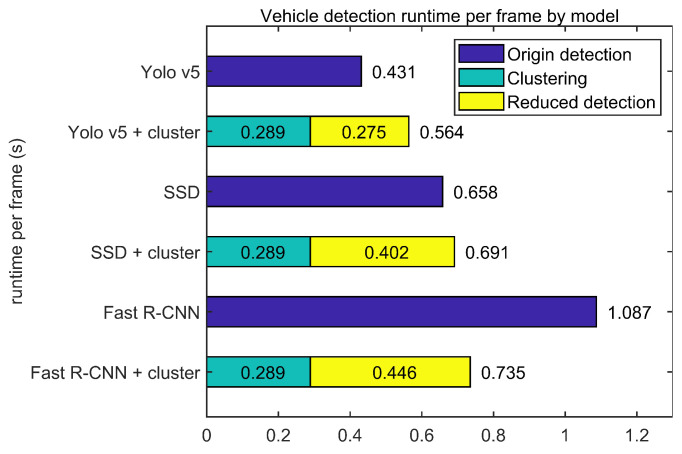
Detection runtime per frame by model on board.

Figure 20 shows the processing time per frame of the model using the three 2D image detector models and the proposed method, respectively. In an embedded board, the clustering and preprocessing process of the image took 0.289 s per frame. Each model was post-processed by a reduced model with a region proposal process omitted, where Yolo v5 reduced by about 36.2%, SSD reduced by about 38.9%, and Fast R-CNN reduced by about 58.9%. The resulting total execution time increased by about 30.8% for Yolo v5 and 38.9% for SSD, but decreased by about 32.3% for Fast R-CNN. For the one-stage detectors, Yolo v5 and SSD, the detector’s runtime reduction was about 37%, which was small compared to the added clustering load, resulting in an increase in total runtime. Conversely, Fast R-CNN, a two-stage detector, decreased the total runtime due to the large load reduction in the detector compared to the clustering load added with the detector’s runtime reduction of about 58.9%.

## 6. Conclusions

In this paper, we proposed a method for clustering point cloud data collected from LiDAR sensors and separating them into individual objects, using their physical characteristics to perform primary semantic detection using a machine learning-based classifier to generate individual object image frames and replace the box prediction of conventional 2D CNN detection. We performed object detection on the KITTI dataset with 1.92% higher accuracy than YOLO v5, an existing 2D-based detection algorithm, and up to 8.97% higher accuracy than YOLO v5 in situations with high illumination of the image data and up to 13.58% higher accuracy in situations with low illumination of the camera data. In the process, the execution time increased by about 23.7%.

The proposed technique can benefit from reduced execution time depending on the operating environment of the detection model and the form of the underlying algorithm. The proposed model running on a PC rather than an embedded board gained about 3 frames per second (fps) when compared to the original YOLO v5. In addition, unlike the first-stage detector, YOLO v5, and the SSD, which reduced the execution time of the detection model by about 37%, the second-stage detector, Fast R-CNN, showed a decrease in execution time of about 58.9%, indicating a decrease in the execution time of the entire process, unlike the other two models. This confirms that when the region proposal process is performed sequentially or in a heavily loaded model, the proposed method can achieve the effect of reducing execution time in addition to improving accuracy.

Object detection algorithms based on 2D images collected by cameras have high accuracy under ideal optical conditions, but in environments with poor visibility, they are less accurate than algorithms based on 3D data. In this case, if physical characteristics that are independent of the optical environment can be obtained from 3D data to form ROIs that provide high reliability from 2D images, 2D-image-based object detection algorithms can add the characteristic advantages of 3D-based algorithms. In this process, clustering and machine learning-based classification of 3D data can be used to achieve lower data throughput than 3D data-based CNN object detection algorithms.

However, because the proposed technique uses a classifier that learns physical properties based on a single object called a vehicle, it is necessary to explore other suitable physical properties and redesign the classification model in order to classify other objects with similar physical properties. In addition, it is a method of performing an object detection algorithm by additionally processing LiDAR data and extracting primary semantic detection results as individual images in the 2D image detection process, so it has a disadvantage compared to general 2D detection algorithms in the memory consumption area. Therefore, based on these findings, we will continue to explore physical properties and classification methods suitable for other major objects, enabling universal object detection and reducing memory usage by making 3D data processing lighter.

## Figures and Tables

**Table 1:** Experimental parameters used to train and validate the algorithm.

Dataset Parameter	Value
Train set	7518 instances
Validation set	1000 instances
Test set	1000 instances
HFOV	360${}^{\circ }$
VFOV	26.8${}^{\circ }$
Point per frame	65,536
Number of channel	64
Point per channel	1024
Model parameter	Value
Classifier	Fine-grain KNN
Distance metrics	Euclidean
Weights	Same
Normalize	True
Training parameters	7

## Data Availability

Not applicable.

## Abbreviations

The following abbreviations are used in this manuscript:

LiDAR	Light Detect and Ranging
GPU	Graphics Processing Unit
CNN	Convolution Neural Network
ROI	Region of Interest
KNN	K-Nearest Neighborhood
PCL	Point Cloud Library
YOLO	You Only Look Once
